# Epidemiological Situation of Antibiotic-Resistant Microorganisms Identified in Patients Hospitalised at the University Teaching Hospital in Bialystok, Poland, in the 2020–2023 Period

**DOI:** 10.3390/antibiotics14111128

**Published:** 2025-11-07

**Authors:** Monika Filipkowska, Magda Orzechowska, Mateusz Zarychta, Mateusz Cybulski

**Affiliations:** 1 Department of Epidemiology, District Sanitary and Epidemiological Station in Bialystok, 15-062 Bialystok, Poland; 2 Department of Integrated Medical Care, Faculty of Health Sciences, Medical University of Bialystok, 15-096 Bialystok, Poland; magda.orzechowska@umb.edu.pl (M.O.); mateusz.cybulski@umb.edu.pl (M.C.); 3 Faculty of Technical Physics, Information Technology and Applied Mathematics, Lodz University of Technology, 93-005 Lodz, Poland; 251443@edu.p.lodz.pl

**Keywords:** antibiotic-resistant microorganisms, antimicrobial resistance, hospital-acquired infections

## Abstract

Introduction: Antimicrobial resistance constitutes one of the key challenges to public health. It is a particularly serious problem in the context of hospital-acquired infections (HAIs). By causing infections that are difficult to treat, multiple-drug-resistant bacteria in the hospital environment often require the use of toxic and costly drugs and prolonged hospital stays, and they result in long-term health consequences for patients, including a high risk of death. This study aimed to assess the epidemiological situation of antibiotic-resistant microorganisms in patients hospitalised at the University Teaching Hospital in Bialystok, Poland, between 2020 and 2023. Methods: Data from epidemiological reports covering 15 alert pathogens, including MRSA, VRE, KPC (+), and ESBL (+), were analysed. Their prevalence was assessed in three groups of wards: intensive care, surgical, and non-surgical. Infection and microbiological testing rates were referenced to the number of hospitalisations and patient-days. Results: A total of 6066 cases of infections caused by resistant microorganisms were identified. The most frequently isolated pathogen was *Enterococcus faecium* VRE, peaking in 2022 (11.43 per 1000 patients). A marked increase in *Klebsiella* spp. KPC (+) and *Enterobacter* spp. ESBL (+) was observed, particularly in the 2021–2022 period. Intensive care units showed the highest infection rate (30–36 per 1000 patient-days). In the 2022–2023 period, infections detected within <72 h of admission predominated, which may indicate prior patient colonisation or intensified screening. Conclusions: The rise in infections caused by antibiotic-resistant bacteria requires a high level of microbiological surveillance to be maintained, especially in intensive care units, and preventive measures at hospital admission to be strengthened.

## 1. Introduction

Antimicrobial resistance (AMR)—the ability of a microorganism to withstand the bactericidal action of antibiotics—is one of the most serious public health challenges, threatening a return to an era when common and currently potentially mild infections carried a high risk of death [1,2].

The study period (2020–2023) coincided with the COVID-19 pandemic, which significantly impacted hospital operations worldwide. During this time, healthcare systems experienced disruptions in routine patient admissions, resource reallocation, and changes in infection control processes. Studies have shown that the pandemic influenced both the dynamics of hospital admissions and the epidemiology of nosocomial infections, including increased rates of antimicrobial resistance and changes in antibiotic use patterns [3,4].

Antimicrobial resistance is a serious problem in hospital environments, particularly in the context of hospital-acquired infections (HAIs). Healthcare facilities present specific conditions conducive to the transmission of drug-resistant microorganisms, including the presence of immunocompromised patients, the necessity of using antibiotics (including broad-spectrum agents), the application of invasive medical procedures, and constant contact between medical staff, patients, and medical equipment [5,6]. Multiple-drug-resistant bacteria such as methicillin-resistant *Staphylococcus aureus* (MRSA), vancomycin-resistant *Enterococcus* (VRE), carbapenemase-producing *Klebsiella pneumoniae* (KPC), and strains producing extended-spectrum beta-lactamases (ESBL) are particularly dangerous to humans as they cause infections that are difficult to treat and often require the use of potent, toxic, and costly drugs, leading to prolonged hospitalisation and, in some cases, patient death [6]. The GBD 2021 Antimicrobial Resistance Collaborators study (2024), coordinated by the Institute for Health Metrics and Evaluation at the University of Washington, found that in 2021, antimicrobial resistance was associated with 4.7 million deaths, with 1.1 million of them directly attributable to AMR. Without preventive action, this number could rise to 1.9 million per year by 2050. The authors emphasise the need for prevention, the development of next-generation drugs, and the reduction in inappropriate antibiotic use [7]. According to data from the Point Prevalence Survey conducted by the European Centre for Disease Prevention and Control [8], in the 2022–2023 period, approximately 4.3 million patients in hospitals across the European Union and the European Economic Area acquired at least one HAI annually. HAIs were associated with over 90,000 deaths each year. Meanwhile, US CDC data (2025) indicate that on any given day, around 1 in 31 hospital patients in the United States has at least one HAI.

The framework for collecting information on hospital-acquired infections and alert pathogens in Poland is established in the Act of 5 December 2008 on the prevention and control of infections and infectious diseases in humans, which requires the directors of healthcare entities providing inpatient services to prepare and submit reports on the current epidemiological situation of the hospital to the competent State Sanitary Inspector [9,10,11,12].

Hospital-acquired infections caused by antibiotic-resistant bacterial strains constitute a growing epidemiological problem in Polish hospitals; therefore, this study aimed to conduct an epidemiological analysis of antibiotic-resistant microorganisms identified in patients requiring hospitalisation in the Department of Anaesthesiology and Intensive Care, in surgical wards, and in non-surgical wards of the University Teaching Hospital in Bialystok, Poland, between 2020 and 2023. Based on this aim, the following research questions were formulated:

1. Which species of antibiotic-resistant microorganisms were most frequently identified in patients hospitalised in the Department of Anaesthesiology and Intensive Care (DAIC), surgical wards, and non-surgical wards of the University Clinical Hospital in Bialystok, Poland, between 2020 and 2023?
2. Are there differences in the frequency of isolation of antibiotic-resistant microorganisms between patients hospitalised in different types of hospital wards (DAIC, surgical, non-surgical)?

## 2. Results

Between 2020 and 2023, a total of 6066 biological samples obtained from patients hospitalised at the University Teaching Hospital in Bialystok, Poland were found to contain one of the microorganisms with the following resistance mechanisms: MRSA, *Enterococcus faecalis* VRE, *Enterococcus faecium* VRE, *Escherichia coli* ESBL (+), *Escherichia coli* KPC (+), *Escherichia coli* MBL/NDM (+), *Klebsiella* spp. ESBL (+), *Klebsiella* spp. KPC (+), *Klebsiella pneumoniae* MBL/NDM, *Enterobacter* spp. ESBL (+), *Enterobacter* spp. KPC (+), *Pseudomonas aeruginosa*, *Acinetobacter* spp., *Clostridium difficile*, *Clostridium perfringens*. The total number of hospitalisations during the study period was nearly 210,000, showing a 1.5-fold increase from 42,893 in 2020 to 61,246 in 2023.

In all years analysed, the dominant alert pathogen detected in the hospital was *Enterococcus faecium* VRE, with the highest incidence recorded in 2022 at 11.4 per 1000 hospitalised patients. The second most frequently identified group of pathogens was *Klebsiella* spp. ESBL (+). In 2022, a significant increase was observed in the number of infections caused by various *Klebsiella* spp. KPC (+) species. In 2020, no infections due to *Klebsiella* spp. KPC (+) were reported; in 2021, there were 4 *Klebsiella* spp. KPC (+) infections, whereas in 2022, the number of *Klebsiella* spp. KPC (+) infections was 48. That same year, there was also a marked increase (from 1 case in 2021 to 32 cases in 2022) in infections caused by *Enterobacter* spp. ESBL (+) strains. Table 1 show the occurrence of the fifteen antibiotic-resistant microorganisms across all wards of the University Teaching Hospital in Bialystok, Poland.

**Figure 1:**
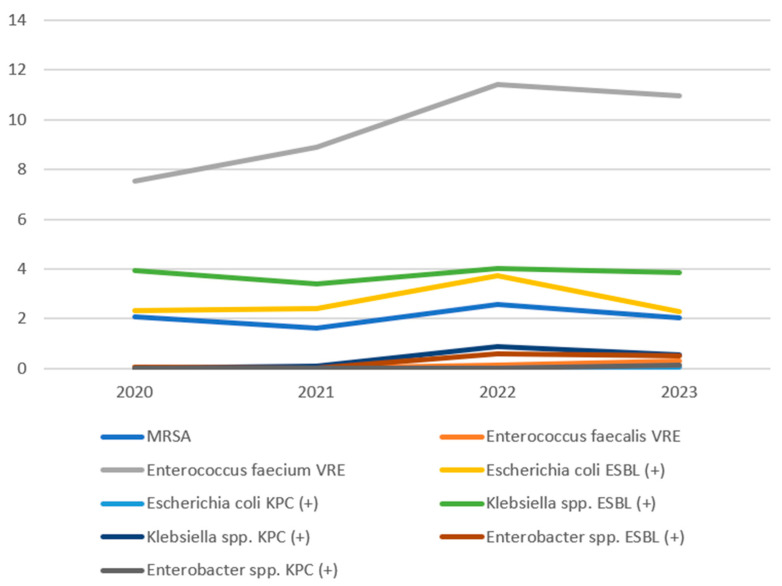
Incidence trends of the studied pathogens.

During the analysed period, the overall infection burden was similar between Gram-positive and Gram-negative bacteria, with a slight predominance of Gram-positive bacteria in the last year of the study. Gram-positive bacteria primarily included MRSA, *Enterococcus faecalis* VRE, and *Enterococcus faecium* VRE. Notably, *E. faecium* VRE dominated in this group, with the isolation rate systematically increasing between 2020 and 2022, reaching peak incidence in 2022 (11.43/1000 patients) and then stabilising in 2023. *Enterococcus faecalis* VRE occurred significantly less frequently, while MRSA maintained a stable, low level throughout the observation period. Gram-negative bacteria constituted the second most common group of alarm pathogens, among which *Klebsiella* spp. (both ESBL (+) and KPC (+)), *Enterobacter* spp. ESBL (+), and *Escherichia coli* ESBL (+) were most frequently isolated. The largest increase in the number of isolations was recorded in the 2021–2022 period, particularly for *Klebsiella* spp. KPC(+) and *Enterobacter* spp. ESBL (+). The share of *E. coli* ESBL (+) remained relatively constant, while *E. coli* KPC (+) and *Enterobacter* spp. KPC (+) strains were rare and detected sporadically (Figure 1).

**Figure 2:**
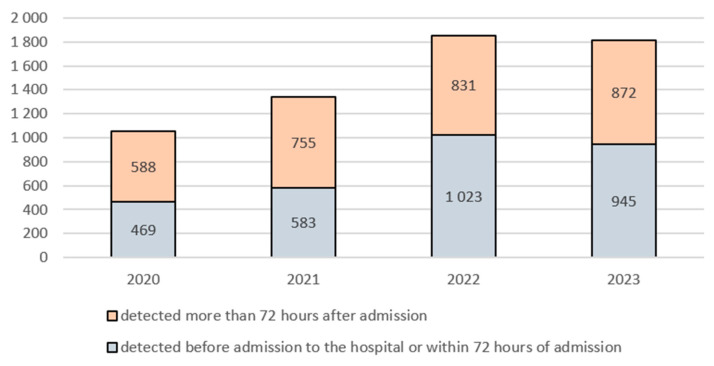
Number of infections detected up to and beyond 72 h after hospital admission by year in the 2020–2023 period.

In 2022, a change was noted in the proportion of infections detected before admission to the hospital or within 72 h of admission, compared to infections detected more than 72 h after admission. This applied to the total number of all analysed bacterial strains. In the 2020–2021 period, infections diagnosed more than 72 h after admission accounted for the greater proportion, whereas in the 2022–2023 period, the situation was reversed, with the vast majority of infections caused by alert bacteria being detected before hospital admission or within 72 h of admission. In 2020, 469 infections were detected within 72 h of admission, and 588 alert microorganisms were identified more than 72 h after admission. In 2022, these numbers were 1023 and 831, respectively (Figure 2).

The epidemiological analysis of bacterial resistant strains, taking into account the number of patient-days in the ward, the number of microbiological tests performed, and the number of multiple-drug-resistant bacteria detected in specific ward profiles (anaesthesiology and intensive care, surgical wards, and non-surgical wards) in the 2020–2023 period showed that the highest rate of microbiological testing per 1000 patient-days was consistently recorded in the anaesthesiology and intensive care wards. This result reflects the high intensity of diagnostic and epidemiological surveillance of critically ill patients hospitalised in such wards. At the same time, these wards maintained the highest infection rate (an average of 30–36 infections per 1000 patient-days), confirming the considerable infection risk in this group of patients. Surgical wards had the lowest infection rate (3–4.5 per 1000 patient-days), although the relatively low microbiological testing rate may indicate limited microbiological diagnostics, performed mainly when clear signs of infection were present. This requires further analysis in the context of possible incorrect or delayed diagnosis of infections. In non-surgical wards, a systematic increase was observed in both the number of microbiological tests performed and the number of infections detected, translating into an increase in the infection rate from 5.55 to 9.18 per 1000 patient-days between 2020 and 2022. In 2023, there was a slight decrease in this rate (8.68 per 1000 patient-days), which may indicate either a stabilisation of the situation or the effectiveness of implemented anti-epidemic measures. Detailed data are presented in Table 2.

## 3. Discussion

Between 2020 and 2023, a total of 6066 antibiotic-resistant microorganisms were identified at the University Teaching Hospital in Bialystok, Poland, as included in the present analysis. This finding provides clear evidence of the problem of antimicrobial resistance in hospital settings. This figure corresponds to the population of a typical small town in Poland, which vividly illustrates the scale of the epidemiological threat faced by the healthcare facility during this period. The problem of antimicrobial resistance is part of the broader context of hospital-acquired infections (HAIs), as evidenced, for example, by the meta-analysis by Raoofi et al. [13], which aimed to assess the global prevalence of HAIs. The authors analysed data from 400 studies published between 2000 and 2021, covering over 29 million patients from various regions of the world, and demonstrated that the average global prevalence of HAIs is 14% and shows an upward trend of 0.06% per year [13].

The latest national analyses conducted by the Chief Sanitary Inspectorate confirm the increase in the isolation of antibiotic-resistant strains observed in our analysis during the COVID-19 pandemic, including carbapenemase-producing *Klebsiella pneumoniae* (KPC), ESBL-positive *Escherichia coli*, and vancomycin-resistant *Enterococcus faecium* (VRE). The report emphasises that these trends intensified between 2020 and 2022, which is related, among other things, to disruptions in infection control procedures and changes in antibiotic use during the COVID-19 pandemic [14]. This phenomenon reflects the nationwide trend of increasing antimicrobial resistance, confirming the need to maintain constant microbiological surveillance and strengthen rational antibiotic therapy programmes in Polish hospitals [15]. The data obtained in the analysed hospital regarding the microbial structure and resistance mechanisms are similar to the results published by Chmielarczyk et al. (2021), who conducted a four-year analysis of 4218 cases of hospital-acquired bloodstream infections in southern Poland [16]. In their study, Gram-positive bacteria accounted for 70.9% of all isolations, and Gram-negative bacteria for 27.8%, with *Staphylococcus aureus* (including MRSA) and *Enterococcus* spp. (including VRE) dominating, while in the Gram-negative group, *Klebsiella* spp. and *Enterobacter* spp. exhibiting ESBL or KPC mechanisms were the most common [16].

The present analysis demonstrates that the most frequently detected alert pathogen was *Enterococcus faecium* VRE, which reached its highest incidence in 2022 at 11.43 per 1000 hospitalised patients, which may indicate a massive spread of multiple-drug-resistant (MDR) strains during this period. The second most common group of pathogens was *Klebsiella* spp. ESBL (+). In Norway, there was a similarly rapid increase in the prevalence of HAIs caused by *Enterococcus faecium* VRE strains in the 2010–2015 period. Consequently, a random sample of 239 Norwegian blood isolates of *E. faecium* VRE from 2010 to 2015 and 261 isolates of *E. faecium* VRE from 2008 and 2014 were analysed. Whole-genome sequencing of *E. faecium* was performed to determine population structure, the presence of resistance genes, virulence factors, and other elements. This was followed by a comparative analysis with *E. faecium* VRE genomes from around the world, using a set of 272 isolates from 1946 to 2022. The findings showed that the population structure of Norwegian *E. faecium* isolates corresponds to globally dominant clones, particularly the concurrently occurring European types [17].

Based on the results of the present study, a particularly concerning finding was the sharp increase in *Klebsiella* spp. KPC (+) infections—from 0 cases in 2020 to 4 cases in 2021 and as many as 48 cases in 2022, representing a 12-fold increase compared to the previous year. Such a trend indicates the progressive spread of carbapenem resistance mechanisms, which are associated with “last resort” antibiotics (i.e., imipenem, meropenem, doripenem, ertapenem). Between 2021 and 2022, there was a 32-fold increase in cases of *Enterobacter* spp. ESBL (+) infections. This abrupt rise may reflect the emergence of epidemic outbreaks or improved detection due to intensified epidemiological surveillance.

As described in the literature, a study of *Klebsiella pneumoniae* isolates from outpatients in Belgrade (Serbia) revealed that the prevalence of carbapenemases is comparable to their distribution in other European regions. The dominant enzyme in that study was OXA-48, which may indicate a shift in epidemiological trends, as NDM had previously been most frequently detected. These results suggest possible transformations in the molecular epidemiology of resistance and highlight the need for further studies [18].

It is also noteworthy that in the present analysis a change was observed in the temporal pattern of infection detection. In the 2020–2021 period, infections detected more than 72 h after hospital admission predominated, indicating the typical nature of HAIs. However, by the 2022–2023 period, the situation had reversed, with the vast majority of cases being detected before hospital admission or within the first 72 h of hospitalisation, suggesting

- an increase in admissions of patients already colonised or infected with alert microorganisms (e.g., transferred from other long-term care facilities);
- increased vigilance and intensified screening upon admission, partly as a consequence of the experience gained during the SARS-CoV-2 pandemic [19];
- possible underestimation of the exposure time to the microorganism due to delayed onset of symptoms.

This change warrants further analysis, particularly in the context of preventing the transmission of alert pathogens between different healthcare facilities.

A review of current scientific reports confirms the growing problem of patient colonisation with microorganisms exhibiting multiple drug resistance (MDR). A study conducted among Greek patients between 2019 and 2022 showed that, out of 4370 patients hospitalised in three Greek hospitals, 31.1% were colonised with carbapenem-resistant *Enterobacterales*, 30.1% with carbapenem-resistant *Acinetobacter baumannii*, 5.8% with carbapenem-resistant *Pseudomonas aeruginosa*, and 28.4% with vancomycin-resistant enterococci [20].

The comparison of data from 2020 to 2023 enables an assessment of the epidemiological situation regarding HAIs in relation to the number of patient-days, the number of microbiological tests, and the number of infections detected in the three groups of wards: anaesthesiology and intensive care, surgical wards, and non-surgical wards. In the anaesthesiology and intensive care wards analysed, the rate of microbiological testing remained high compared to surgical and non-surgical wards, oscillating between 1240 and 1330 tests per 1000 patient-days. This means an average of more than one microbiological test per patient on each day of stay. This high intensity of microbiological diagnostics is consistent with the ward profile and the high risk of invasive infections (including those associated with the routine use of ventilators and catheters). At the same time, the infection rate remained high, from 28.29 per 1000 patient-days in 2020 to 35.96 in 2023, peaking in 2021 at 37.53 per 1000 patient-days. These values indicate a significant infection risk in critically ill patients and confirm the need for continued close microbiological surveillance and maintenance of infection control systems.

A study conducted in the intensive care unit of a tropical diseases hospital in Ho Chi Minh City, Vietnam, between 2014 and 2016 assessed the incidence of colonisation and infection with antibiotic-resistant bacteria among adult patients hospitalised for more than 48 h. Of the 364 patients, 61.3% were colonised with resistant bacteria, most commonly ESBL (+) *E. coli* and *Klebsiella* spp. (based on rectal swabs), carbapenemase-producing *Acinetobacter* spp. (based on respiratory tract samples), and MRSA (based on nasal swabs). The colonisation rate was 9.8 per 100 patient-days. Hospital-acquired infections occurred in 23.4% of patients, most frequently as pneumonia, urinary tract infections, and bloodstream infections, with more than half of these infections being preceded by colonisation with the same pathogen. The main risk factors for colonisation with multiple-drug-resistant bacterial strains, according to the authors of this prospective observational study in ICU patients in Vietnam, were comorbidities and the use of vascular catheters. The conclusions underline the need for preventive measures such as colonisation monitoring, hand hygiene, rational antibiotic use, and isolation of colonised patients to limit the spread of resistant bacteria [21].

The analysis of the epidemiological situation in surgical wards in the present study shows that the number of patient-days in these wards increased systematically during the study period, which may result from the restoration of surgical activity after restrictions resulting from the COVID-19 pandemic. The rate of microbiological testing in these types of wards increased from 203.59 per 1000 person-days (2020) to 227.81 per 1000 person-days (2023), which may indicate gradually improving patient care in terms of microbiological diagnostics. Nevertheless, the infection rate remained low compared to anaesthesiology and intensive care and non-surgical wards, fluctuating between 3.60 and 4.59 per 1000 patient-days. While this may indicate effective prevention of surgical site infections, the low proportion of tests relative to patient-days may suggest a risk of missed early or accurate infection diagnosis, particularly in the absence of clear clinical symptoms.

Data from a 2023 study by Erdem et al. [22] indicate that factors significantly associated with mortality due to opportunistic infections include urinary catheterisation, urgent surgery, heart valve replacement, advanced age, and repeated surgical interventions within 30 days after surgical site infection. In that study, Gram-positive bacteria accounted for 43.1% of infections, while Gram-negative bacteria were responsible for 27.5%. Staphylococci predominated among Gram-positive bacteria; the most commonly isolated among Gram-negative bacteria were *Escherichia coli*, *Pseudomonas aeruginosa*, and *Klebsiella* spp. [22].

The assessment of the epidemiological situation in the non-surgical wards of the University Teaching Hospital in Bialystok, Poland, revealed that these wards generated the highest number of patient-days compared to anaesthesiology and intensive care and surgical wards, reflecting the scale of hospitalisations and the length of patient stays. Between 2020 and 2022, a steady increase in the infection rate was observed, from 5.55 to 9.18 per 1000 patient-days, accompanied by an increase in the intensity of microbiological testing from 358.34 to 405.81 per 1000 patient-days. This increase may indicate both improved infection detection and a genuine rise in their prevalence, particularly in the context of the COVID-19 pandemic and the deterioration in the general health of patients with chronic non-communicable diseases. In 2023, a slight decrease in the infection rate to 8.68 per 1000 patient-days was recorded, along with a reduction in the number of tests performed. This may suggest an improvement in the occurrence of opportunistic infections or a decrease in diagnostic vigilance towards patients.

In light of the increases in the isolation of resistant pathogens observed in our analysis, it is necessary to strengthen efforts in the areas of both rationalisation of antibiotic therapy (antimicrobial stewardship, ASP) and infection prevention and control (IPC) in Polish conditions. In Poland, research has shown that antibiotic consumption remains relatively high, and the implementation of ASP in hospitals still requires strengthening [23,24]. At the same time, assessments of compliance with hand hygiene standards and other IPC procedures indicate significant gaps—for example, compliance with the “bare below the elbow” principle and the availability of procedures across departments remain inconsistent [25]. Therefore, we recommend that the results of our study be used as an impetus for: systematic monitoring and reduction in broad-spectrum antibiotic use, the introduction of audits and control of antibiotic consumption in high-risk departments, and the intensification of IPC activities, including educational programmes in this area.

The increase in infections detected shortly after admission (<72 h) may be the result of several interacting factors:

- an increase in the number of patients admitted with pre-existing colonisation or infection, e.g., transferred from long-term care facilities or other facilities to the hospital;
- intensified screening following the COVID-19 pandemic and the diagnosis of carrier status/colonisation;
- changes in the structure of admissions—a higher percentage of patients with chronic conditions, previously hospitalised or receiving outpatient treatment;
- changes in the functioning of the healthcare system related to the pandemic (e.g., delays in care, changes in the hospital admission process, staffing limits) may have affected the clinical condition of patients and the hospital environment.

The literature indicates that the COVID-19 pandemic has influenced the epidemiology of nosocomial infections and the colonisation of multidrug-resistant pathogens [26,27,28,29]. Our observations of a significant increase in the isolation of *Klebsiella* spp. strains with the KPC(+) mechanism in the 2021–2022 period and the increasing incidence of ESBL(+) *Enterobacter* spp. are consistent with the results of a recent analysis conducted in an intensive care unit comparing the pandemic and post-pandemic periods of COVID-19. Golli et al. [30] found a significant increase in the incidence of multidrug-resistant Gram-negative bacteria, especially *Klebsiella* spp. and *Acinetobacter* spp., as well as increased bacterial resistance to last-line drugs such as colistin and tigecycline in the post-pandemic period.

## 4. Study Limitations

The limitations of the study primarily include the use of secondary data derived from epidemiological reports prepared by the hospital, which limits control over their quality and completeness. The lack of access to patients’ clinical data prevents the analysis of risk factors, treatment course, and therapeutic outcomes, which could have deepened the interpretation of the results. Another limitation is the absence of molecular analysis of the pathogens, which prevents identification of the sources of transmission and possible epidemic outbreaks. Furthermore, the classification of infections as hospital-acquired or pre-existing (detected within 72 h of admission) may have been subject to error due to delayed onset of symptoms, which affects the interpretation of the infection source. Moreover, the lack of access to data on antibiotic consumption (denominator) in the studied period limits the possibility of assessing the role of the use of specific antibiotic classes as factors determining resistance in the studied population.

## 5. Materials and Methods

### 5.1. Study Design

We analysed the Annual Reports on the occurrence of alert pathogens at the University Teaching Hospital in Bialystok, Poland between 2020 and 2023, prepared by the hospital in accordance with the legal requirements of the Act of 5 December 2008 on the prevention and control of infections and infectious diseases in humans and the Regulation of the Minister of Health of 23 December 2011 on the list of alarm factors, registers of hospital infections and alarm factors and reports on the current epidemiological situation of the hospital for the purpose of epidemiological surveillance conducted by the State District Sanitary Inspector in Bialystok, Poland. This study used selected data from the Annual Reports, including the number of hospitalised patients per year in individual wards, the number of patient-days per year in individual wards, the number of microbiological tests performed per year in individual wards, and the number of patients in whom a specific antibiotic-resistant microorganism was detected in a given year.

An alarm pathogen is defined in Polish law as a pathogenic microorganism which, due to its potential for rapid spread and/or significant resistance to antimicrobial agents, constitutes an epidemiological threat and requires constant monitoring and reporting.

The analysis of the occurrence of drug-resistant microorganisms across all hospital wards covered 15 pathogens with the following antimicrobial resistance mechanisms: MRSA, *Enterococcus faecalis* VRE, *Enterococcus faecium* VRE, *Escherichia coli* ESBL (+), *Escherichia coli* KPC (+), *Escherichia coli* MBL/NDM (+), *Klebsiella* spp. ESBL (+), *Klebsiella* spp. KPC (+), *Klebsiella pneumoniae* MBL/NDM, *Enterobacter* spp. ESBL (+), *Enterobacter* spp. KPC (+), *Pseudomonas aeruginosa*, *Acinetobacter* spp., *Clostridium difficile*, *Clostridium perfringens*.

Only for *Klebsiella pneumoniae* MBL/NDM, the available data enabled differentiation between clinical infection and colonisation—patients with symptomatic *Klebsiella pneumoniae* MBL/NDM infection were distinguished from those colonised with *Klebsiella pneumoniae* MBL/NDM in a given ward.

Furthermore, in the available epidemiological surveillance reports, the total number of patients with an identified alert pathogen was divided into patients whose microbiological testing (i.e., microorganism identification) was performed within 72 h of hospital admission, and patients in whom the infection was diagnosed more than 72 h after admission.

Each patient was counted once per year for each pathogen (if a given pathogen was isolated multiple times from the same individual within a single year, only the first isolation of that pathogen in that year was included for that patient). Data consistency verification against annual epidemiological reports involved comparing the arithmetic sums of patients infected with a specific pathogen with the corresponding annual summaries at both the hospital ward and yearly levels.

The epidemiological situation was further analysed in wards with specific profiles, i.e., the Department of Anaesthesiology and Intensive Care, surgical wards associated with general surgery, cardiac surgery, vascular surgery, neurosurgery, oncological surgery, otolaryngology, dental surgery, maxillofacial surgery, urology, plastic and reconstructive surgery, and others, excluding orthopaedics, as well as non-surgical wards, including internal medicine, cardiology, nephrology, neurology, rheumatology, endocrinology, pulmonology, and others, excluding haematology and oncology. In these wards, the following parameters were assessed: the number of patient-days, the microbiological testing rate, and the frequency of infections caused by the fifteen aforementioned antibiotic-resistant microorganisms.

### 5.2. Ethical Issues

This study used secondary anonymised data provided by the State District Sanitary Inspector in Bialystok, Poland upon request for public information. Therefore, approval from the Bioethics Committee of the Medical University of Bialystok, Poland was not required.

### 5.3. Statistical Analysis

Statistical analysis was carried out using MS Excel and was based on quantitative data, i.e., the number of cases of individual infections with microorganisms included in the reports, and the calculation of epidemiological measures including incidence per 1000 patients for individual pathogens, overall incidence per 1000 patients, testing rate per 1000 patient-days (DAIC, surgical wards, non-surgical wards), and the infection rate per 1000 patient-days (DAIC, surgical wards, non-surgical wards), which were calculated as follows:

Incidence per 1000 patients for individual pathogens—the number of patients in whom a given alert pathogen occurred in a given year, divided by the total number of patients in that year, expressed per 1000 patients.

Overall incidence per 1000 patients—the number of patients in whom any of the alert pathogens occurred in a given year, divided by the total number of patients in that year, expressed per 1000 patients.

Testing rate per 1000 patient-days—the number of microbiological tests performed in relation to 1000 patient-days in specific ward profiles (DAIC, surgical wards, non-surgical wards).

Infection rate per 1000 patient-days—the number of detected infections (total infections caused by the 15 antibiotic-resistant microorganisms) in relation to 1000 patient-days in specific ward profiles (DAIC, surgical wards, non-surgical wards).

The number of patient-days (as defined in public statistics) is the total number of days of hospital stay of all patients in the study period, with the day of admission and the day of discharge counted as one day.

The results are presented as proportions with corresponding 95% confidence intervals (95% CI).

## 6. Conclusions

1. At the University Teaching Hospital in Bialystok, Poland, there is a rising threat associated with the spread of multiple-drug-resistant microorganisms, particularly VRE, ESBL (+), and KPC (+).
2. The change in the infection profile (increase in early infections) indicates the need for broader patient monitoring upon admission and analysis of the sources of their infections.
3. The sharp increase in cases of *Klebsiella* spp. KPC (+) and *Enterobacter* spp. ESBL (+) may indicate the emergence of epidemic outbreaks or improved detection as a result of intensified epidemiological surveillance.
4. The intensive care unit remains the greatest challenge from the perspective of hospital infection control and requires maintaining high-intensity testing and preventive measures.
5. In surgical wards, low infection rates may be the result of effective prevention; however, there is a risk of underdiagnosis—regular evaluation of microbiological testing practices is needed.
6. In non-surgical wards, the rising trend in infection detection indicates increased vigilance and improved microbiological surveillance but requires ongoing monitoring and optimisation of anti-epidemic procedures.
7. Further measures should include monitoring resistance trends, analysing infection outbreaks, and assessing the effectiveness of implemented anti-epidemic interventions.
8. Based on the results, we recommend the following in clinical practise:
  - implementing targeted screening for patients admitted from high-risk groups (transfers, long-term care stays);
  - strengthening surveillance of *Enterobacter* spp. ESBL (+) and *Klebsiella* spp. KPC (+);
  - continuing and expanding programmes to combat antibiotic resistance, with particular focus on reducing the use of carbapenems and broad-spectrum cephalosporins;
  - maintaining high-intensity monitoring in intensive care units.

## Figures and Tables

**Table 1:** Number of infections by individual pathogens and incidence per 1000 patients in 2020–2023 period.

	Year 2020					Year 2021					Year 2022					Year 2023				
	Number of Infected Patients			Incidence ***	95% CI ****	Number of Infected Patients			Incidence ***	95% CI ****	Number of Infected Patients			Incidence ***	95% CI ****	Number of Infected Patients			Incidence ***	95% CI ****
	Total	<72 h *	>72 h **			Total	<72 h *	>72 h **			Total	<72 h *	>72 h **			Total	<72 h *	>72 h **		
MRSA	89	56	33	2.07	(1.68, 2.55)	84	54	30	1.64	(1.32, 2.03)	140	92	48	2.56	(2.17, 3.02)	124	86	38	2.02	(1.69, 2.41)
*Enterococcus faecalis* VRE	2	2	0	0.05	(0.01, 0.18)	0	0	0	0.00	NA	7	7	0	0.13	(0.06, 0.26)	19	10	9	0.31	(0.19, 0.48)
*Enterococcus faecium* VRE	324	175	149	7.55	(6.77, 8.41)	455	222	233	8.90	(8.12, 9.75)	624	374	250	11.43	(10.57, 12.35)	671	357	314	10.95	(10.16, 11.81)
*Escherichia coli* ESBL (+)	100	48	52	2.33	(1.91, 2.83)	123	68	55	2.40	(2.01, 2.87)	204	125	79	3.74	(3.25, 4.28)	139	91	48	2.27	(1.92, 2.67)
*Escherichia coli* KPC (+)	0	0	0	0.00	NA	0	0	0	0.00	NA	0	0	0	0.00	NA	3	1	2	0.05	(0.01, 0.15)
*Escherichia coli* MBL/NDM (+)	1	0	1	0.02	(0.003, 0.16)	6	5	1	0.12	(0.05, 0.26)	11	4	7	0.20	(0.11, 0.36)	4	2	2	0.06	(0.02, 0.17)
*Klebsiella* spp. ESBL (+)	169	52	117	3.94	(3.38, 4.57)	174	59	115	3.40	(2.93, 3.94)	219	112	107	4.01	(3.51, 4.57)	236	98	138	3.85	(3.39, 4.37)
*Klebsiella* spp. KPC (+)	0	0	0	0.00	NA	4	1	3	0.08	(0.02, 0.20)	48	19	29	0.88	(0.66, 1.16)	34	22	12	0.55	(0.39, 0.77)
*Klebsiella pneumoniae* MBL/NDM infection	91	36	55	2.12	(1.72, 2.60)	138	30	108	2.70	(2.28, 3.18)	150	59	91	2.75	(2.34, 3.22)	83	27	56	1.35	(1.09, 1.68)
*Klebsiella pneumoniae* MBL/NDM colonisation	131	54	77	3.05	(2.57, 3.62)	141	67	74	2.76	(2.33, 3.25)	103	55	48	1.89	(1.55, 2.28)	109	67	42	1.78	(1.47, 2.14)
*Enterobacter* spp. ESBL (+)	3	3	0	0.07	(0.02, 0.21)	1	1	0	0.02	(0.002, 0.13)	32	24	8	0.59	(0.41, 0.82)	30	12	18	0.49	(0.34, 0.70)
*Enterobacter* spp. KPC (+)	0	0	0	0.00	NA	0	0	0	0.00	NA	1	0	1	0.02	(0.002, 0.13)	9	5	4	0.15	(0.07, 0.28)
*Pseudomonas aeruginosa*	41	6	35	0.95	(0.70, 1.29)	44	12	32	0.86	(0.64, 1.15)	63	14	49	1.15	(0.90, 1.47)	72	23	49	1.17	(0.93, 1.48)
*Acinetobacter* spp.	47	14	33	1.09	(0.82, 1.45)	73	14	59	1.43	(1.13, 1.79)	77	16	61	1.41	(1.12, 1.76)	119	23	96	1.94	(1.62, 2.32)
*Clostridium difficile*	56	20	36	1.30	(1.00, 1.69)	90	45	45	1.76	(1.43, 2.16)	167	116	51	3.06	(2.62, 3.55)	161	118	43	2.63	(2.25, 3.06)
*Clostridium perfringens*	3	3	0	0.07	(0.02, 0.21)	5	5	0	0.10	(0.04, 0.23)	8	6	2	0.15	(0.07, 0.29)	4	3	1	0.06	(0.02, 0.17)
Total	1057	469	588	24.64	(23.21, 26.15)	1338	583	755	26.17	(24.82, 27.59)	1854	1023	831	33.96	(32.47, 35.51)	1817	945	872	29.67	(28.35, 31.04)

Abbreviations: * < 72 h after hospital admission, ** > 72 h after hospital admission, *** incidence per 1000 patients, **** 95% Confidence Interval for incidence.

**Table 2:** Summary of the number of patient-days, microbiological tests, and infections by antibiotic-resistant strains in relation to ward profile in the 2020–2023 period.

Type of Hospital Ward		Year			
	Parameters	2020	2021	2022	2023
Anaesthesiology and intensive care	Number of person-days	7493	8127	5984	6007
	Number of microbiological tests	9765	10,794	7432	7615
	Testing rate per 1000 person-days	1303.22	1328.17	1241.98	1267.69
	Number of infections detected	212	305	206	216
	Infection rate per 1000 person-days	28.29	37.53	34.43	35.96
Surgical	Number of person-days	45,413	49,493	56,847	59,410
	Number of microbiological tests	9246	8244	11,861	13,534
	Testing rate per 1000 person-days	203.59	166.57	208.65	227.81
	Number of infections detected	207	178	261	217
	Infection rate per 1000 person-days	4.56	3.60	4.59	3.65
Non-surgical	Number of person-days	86,616	96,783	102,951	101,711
	Number of microbiological tests	31,038	38,435	41,779	35,335
	Testing rate per 1000 person-days	358.34	397.13	405.81	347.41
	Number of infections detected	481	698	945	883
	Infection rate per 1000 person-days	5.55	7.21	9.18	8.68

## Data Availability

The original contributions presented in this study are included in the article. Further inquiries can be directed to the corresponding author.
